# Widespread epigenomic, transcriptomic and proteomic differences between hip osteophytic and articular chondrocytes in osteoarthritis

**DOI:** 10.1093/rheumatology/key101

**Published:** 2018-05-08

**Authors:** Julia Steinberg, Roger A Brooks, Lorraine Southam, Sahir Bhatnagar, Theodoros I Roumeliotis, Konstantinos Hatzikotoulas, Eleni Zengini, J Mark Wilkinson, Jyoti S Choudhary, Andrew W McCaskie, Eleftheria Zeggini

**Affiliations:** 1 Wellcome Trust Sanger Institute, Wellcome Trust Genome Campus, Cambridge, UK; 2 Cancer Research Division, Cancer Council NSW, Sydney, NSW, Australia; 3 Division of Trauma & Orthopaedic Surgery, Department of Surgery, University of Cambridge, Addenbrooke’s Hospital, Cambridge, UK; 4 Wellcome Trust Centre for Human Genetics, University of Oxford, Oxford, UK; 5 Lady Davis Research Institute, Jewish General Hospital, McGill University, Montreal, Quebec, Canada; 6 Department of Epidemiology, Biostatistics and Occupational Health, McGill University, Montreal, Quebec, Canada; 7 Department of Oncology and Metabolism, University of Sheffield, Sheffield, UK; 8 Dromokaiteio Psychiatric Hospital of Athens, Chaidari, Athens, Greece

**Keywords:** chondrocyte, osteophyte, epigenomics, transcriptomics, proteomics, genomics

## Abstract

**Objectives:**

To identify molecular differences between chondrocytes from osteophytic and articular cartilage tissue from OA patients.

**Methods:**

We investigated genes and pathways by combining genome-wide DNA methylation, RNA sequencing and quantitative proteomics in isolated primary chondrocytes from the cartilaginous layer of osteophytes and matched areas of low- and high-grade articular cartilage across nine patients with OA undergoing hip replacement surgery.

**Results:**

Chondrocytes from osteophytic cartilage showed widespread differences to low-grade articular cartilage chondrocytes. These differences were similar to, but more pronounced than, differences between chondrocytes from osteophytic and high-grade articular cartilage, and more pronounced than differences between high- and low-grade articular cartilage. We identified 56 genes with significant differences between osteophytic chondrocytes and low-grade articular cartilage chondrocytes on all three omics levels. Several of these genes have known roles in OA, including *ALDH1A2* and cartilage oligomeric matrix protein, which have functional genetic variants associated with OA from genome-wide association studies. An integrative gene ontology enrichment analysis showed that differences between osteophytic and low-grade articular cartilage chondrocytes are associated with extracellular matrix organization, skeletal system development, platelet aggregation and regulation of ERK1 and ERK2 cascade.

**Conclusion:**

We present a first comprehensive view of the molecular landscape of chondrocytes from osteophytic cartilage as compared with articular cartilage chondrocytes from the same joints in OA. We found robust changes at genes relevant to chondrocyte function, providing insight into biological processes involved in osteophyte development and thus OA progression.

**Rheumatology key messages**

- Significant cross-omics differences between osteophytic and low-grade articular chondrocytes identified for 56 genes.
- Genes with cross-omics differences include *ALDH1A2* and *COMP*; both contain genetic variants associated with osteoarthritis.

## Introduction

OA is a degenerative joint disease characterized clinically by pain and loss of physical function [1]. It is very common, affecting >40% of individuals over the age of 70 years [2]. There is no curative therapy; end-stage disease is treated by joint replacement surgery. This procedure provides an opportunity to directly examine and characterize disease tissue from patients using genomic technologies.

A key feature of OA is cartilage degeneration, and several genomics studies have investigated the molecular characteristics of this process (e.g. reviewed in [3]). However, another important feature that can develop in joints affected by OA is the osteophyte, an area of apparent new tissue formation consisting of a cartilage-topped bony outgrowth. Osteophytes can have a significant clinical impact on both pain and loss of movement and are a typical radiographic feature of OA [4]. Osteophytes arise primarily on the margins of the articular cartilage from cells of the periosteum or synovium by a process of endochondral ossification within newly forming fibrocartilage [5]. While the pathogenesis of osteophytes has been studied in mice [5], there is still much to be learned from the molecular characterization of these important structures, particularly in human joints.

A previous study of osteophytic cartilage in the knee investigated gene expression using a microarray and suggested large differences compared with ‘macroscopically intact’ articular cartilage [6]. The authors hypothesized that cells in the osteophyte transition between chondrocyte and hypertrophic chondrocyte phenotypes, as opposed to a stable chondrocyte phenotype in articular cartilage.

In this proof-of-concept study, we report the first analysis of hip OA patient tissue using integrated multi-omics across genome-wide DNA methylation, RNA sequencing and quantitative proteomics to obtain a molecular portrait of chondrocytes from the cartilaginous layer of osteophytes. We identify key molecular players linked to this aspect of the disease and its pathogenesis.

## Methods

More details, including references, are given in the supplementary Methods, available at *Rheumatology* online.

### Patients and samples

The study complies with the Declaration of Helsinki. Tissue samples were collected under National Research Ethics approval reference 11/EE/0011, Cambridge Biomedical Research Centre Human Research Tissue Bank, Cambridge University Hospitals, UK. Three samples each were collected from nine patients (six women, three men, age 44–84 years) undergoing hip joint replacement surgery for OA. All patients provided written informed consent before participation. Cartilage tissue was classified macroscopically for each femoral head as: low-grade, with a smooth surface and no obvious evidence of damage or fibrillation; high-grade, with damaged and fibrillated cartilage; and osteophytic, from the cartilaginous layer of osteophytes located mainly around the margins of the articular surface (sample extraction section of the supplementary methods and supplementary Figs S1 and S2, available at *Rheumatology* online). In each zone, a cartilage sample was removed, with subsequent extraction of DNA, RNA and protein. Cartilage from all structural layers was obtained, with care taken to avoid the removal of non-cartilage tissue. Details for histological examination, chondrocyte preparation, as well as extraction of DNA, RNA and protein are described in the supplementary methods, sample extraction section, available at *Rheumatology* online.

### Proteomics

Liquid chromatography -mass spectrometry (LC-MS) analysis was performed on the Dionex Ultimate 3000 UHPLC system coupled with the Orbitrap Fusion Tribrid Mass Spectrometer (Thermo Fisher Scientific, Waltham, MA, USA). Abundance values were normalized by the sum of all protein abundances in a given sample, then log2-transformed and quantile normalized. No protein was detected in only osteophytic or only articular chondrocytes. Hence we restricted the analysis to 4653 proteins that were quantified in all individuals and tissues. Details for sample processing, LC-MS analysis, protein identification and quantification are described in the supplementary methods, proteomics section, available at *Rheumatology* online.

### RNA sequencing

Multiplexed libraries were sequenced on Illumina HiSeq 2000 (Illumina Inc., San Diego, CA, USA; 75 bp paired-end read length) and a cram file was produced for each sample. We obtained transcript-level quantification using salmon 0.7.2 [7]. After quality control, we retained 14 029 genes. Details for sample processing and read quantification are described in the supplementary methods, RNA sequencing section, available at *Rheumatology* online.

### Methylation

Methylation was assayed using the Illumina 450k BeadChip (Illumina Inc., San Diego, CA, USA). The resulting idat files were parsed and QCed using ChAMP [8] in R, yielding 424 705 probes. The probe beta values were normalized using the funnorm method [9] in R, and converted to M-values. Details for Illumina 450k BeadChip processing and quality control are described in the supplementary methods, methylation section, available at *Rheumatology* online.

### Differential analysis

The proteomics, gene expression, and probe methylation differential analyses were carried out using limma [10] in R. We used a within-individual paired sample design, that is, the individual ID as a covariate in the comparison of osteophytic to low-grade, osteophytic to high-grade and high-grade to low-grade tissue. A Benjamini-Hochberg false discovery rate (FDR) was applied to each analysis to correct for multiple testing. Differentially methylated regions (DMRs) were identified using the DMRcate R package [11].

### Principal component analysis

Principal component analyses were carried out using the prcomp function in R for each omics level, based on significant differences between osteophytic and low-grade cartilage at 0.1% FDR.

### Gene identifier mapping

To map Ensembl gene IDs to gene names and vice versa, we used the assignment in Ensembl (downloaded from Ensembl biomart, GRCh38.p7). We only included instances where a unique Ensembl gene ID corresponded to a unique gene name.

### UK Biobank association analysis

We applied MAGMA v1.06 [12] to test the joint association of genetic variants in the 56 genes changed between osteophytic and low-grade articular cartilage on all three molecular levels. We used genetic association data from UK Biobank, including 2396 hospital-diagnosed hip OA cases and 9593 non-OA controls based on ICD 10 and/or 9 codes; controls were not diagnosed with any musculoskeletal disorders, symptoms or signs. Further details of the dataset, including quality control, are described in the supplementary methods, UK Biobank association analysis section, available at *Rheumatology* online.

Each gene was assigned the single nucleotide polymorphisms (SNPs) located between the gene’s start and stop sites based on NCBI 37.3 gene definitions. For each gene, we used the combined statistic based on the sum of SNP log-*P*-values and the lowest SNP *P*-values, as recommended by MAGMA. Linkage disequilibrium (LD) was calculated from a subset of the UK Biobank samples (see supplementary methods, UK Biobank association analysis section, available at *Rheumatology* online). We then tested whether the 56 genes are more associated with OA than expected by chance, correcting for the potentially confounding effects of sample size, gene size, gene density and the inverse of the mean minor allele count in the gene, as well as the log of these variables, as recommended.

### Integrative Gene Ontology gene set analysis

Gene Ontology (GO) [13] biological process and molecular function gene annotations were obtained from Ensembl Biomart. We followed the integrative cross-omics analysis from [14], as described in detail in the supplementary methods, Gene Ontology gene-set analysis section, available at *Rheumatology* online. Briefly, enrichment of each annotation for each of the three omics levels was assessed using a one-sided hypergeometric test, the *P*-values integrated across the three omics levels followed by randomizations. Significance was defined at 5% FDR. We excluded annotations that were enriched in only one of the omics levels, or where fewer than five genes contributed to the enrichment on at least two omics levels.

## Results

### Widespread differences between osteophytic and articular cartilage

First, we examined genome-wide methylation, gene expression and protein abundance differences between osteophytic and low-grade articular cartilage. At each molecular level, we found widespread differences at 0.1% FDR. In particular, we found significant differences in protein abundance for 942 of 4653 proteins (supplementary Table S1, available at *Rheumatology* online), in gene expression for 3601 of 14 029 genes (supplementary Table S2, available at *Rheumatology* online), and 3161 DMRs that overlapped 3277 genes (supplementary Table S3, available at *Rheumatology* online).

**Fig. 1:**
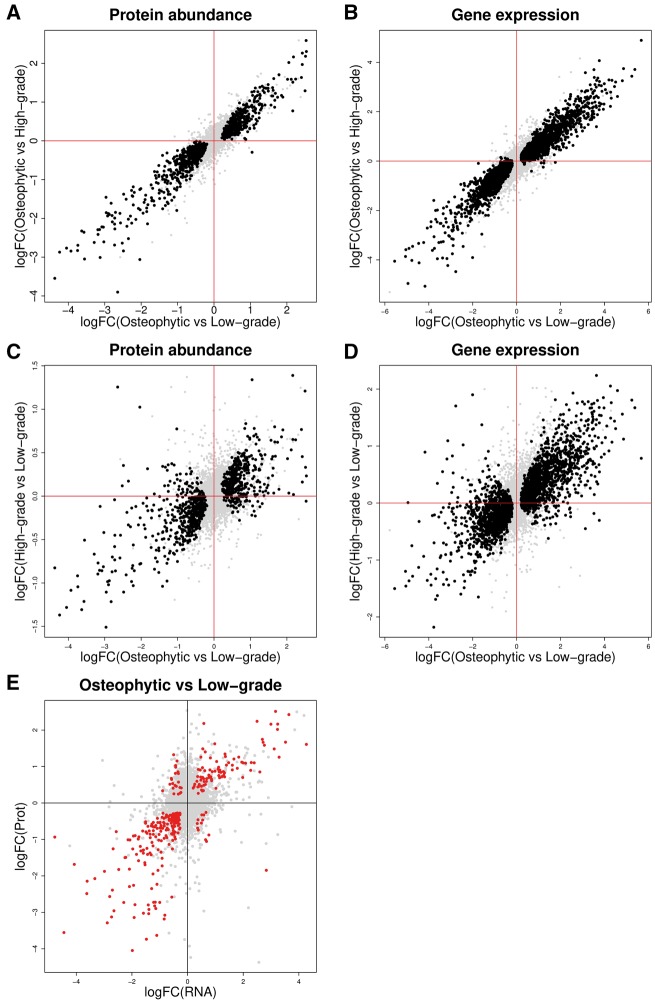
Gene expression and protein abundance differences between osteophytic chondrocytes, low-grade, and high-grade articular chondrocytes (**A** and **B**) Differences between osteophytic and low-grade articular chondrocytes are correlated with differences between osteophytic and high-grade articular chondrocytes for protein abundance (**A**) and gene expression (**B**). (**C** and **D**) Differences between osteophytic and low-grade articular chondrocytes are correlated with differences between high- and low-grade articular chondrocytes for protein abundance (**C**) and gene expression (**D**). (**E**) Differences in gene expression and protein abundance identified between osteophytic and low-grade articular chondrocytes are correlated. Each point represents one gene. Black: genes with significant changes between osteophytic and low-grade articular cartilage at 0.1% FDR. Red: genes with significant changes on both protein and RNA level between osteophytic and low-grade articular chondrocytes at 0.1% FDR.

Second, we also examined molecular differences between chondrocytes from osteophytic and high-grade articular cartilage. Fewer differences were significant at 0.1% FDR: protein abundance differences for 517 of 2653 genes (supplementary Table S1, available at *Rheumatology* online), gene expression differences for 1512 of 14 029 genes (supplementary Table S2, available at *Rheumatology* online) and 1113 DMRs overlapping 1113 genes (supplementary Table S4, available at *Rheumatology* online). However, globally, the gene expression differences between osteophytic and high-grade cartilage were similar to the differences between osteophytic and low-grade articular cartilage (Fig. 1a and b; for estimates of the log-fold-differences in gene expression and protein abundance, the 95% CIs overlap for 97.9% of genes for gene expression and 97.6% of genes for protein abundance). Moreover, for both gene expression and protein abundance levels, of the genes significant at 0.1% FDR in a given comparison (osteophytic/low-grade or osteophytic/high-grade), ⩾99.5% show the same direction of change in the other comparison and ⩾90% are at least nominally significant. Similarly, of the genes contained in DMRs at 0.1% FDR in one comparison, ⩾93.5% are contained in DMRs at 5% FDR in the other comparison.

**Fig. 2:**
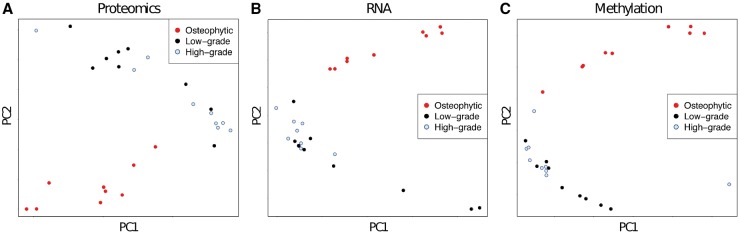
PCA separates osteophytic from low- and high-grade articular cartilage PCA based on proteomics data (**A**), RNA sequencing data (**B**) and probe methylation data (**C**). Each point represents one sample. The PCA was carried out on the proteins or genes with significant differences or within DMRs between osteophytic and low-grade articular cartilage; the plots show that these expression or methylation patterns also separate osteophytic from degraded cartilage. PCA: principal component analysis.

This suggests that the differences between osteophytic and low-grade articular cartilage are similar to, but more pronounced than the differences between osteophytic and high-grade articular cartilage. In agreement with this, on each omics level, we found that a principal component analysis based on the significant differences between osteophytic and low-grade articular cartilage separated the osteophytic cartilage from both the low-grade and the high-grade articular cartilage samples (Fig. 2). Hence we took the comparison of chondrocytes from osteophytic and low-grade articular cartilage as the basis for further analyses.

### Differences between osteophytic and low-grade articular cartilage are correlated with differences between high- and low-grade articular cartilage

At any given level of significance, we identified far more significant differences in the comparison of osteophytic and low-grade articular cartilage than in the comparison of high- and low-grade articular cartilage. For example, at 0.1% FDR, we found no protein with differential abundance between high- and low-grade articular cartilage (supplementary Table S1, available at *Rheumatology* online), only one gene with differential RNA expression (supplementary Table S2, available at *Rheumatology* online), and no differentially methylated regions.

The differences observed between osteophytic and low-grade cartilage are significantly correlated with the differences found between high- and low-grade articular cartilage (Fig. 1c and d; gene expression: Spearman ρ = 0.62, protein abundance: ρ = 0.47; both *P* < 10^−15^). The direction of difference (increase or decrease) in osteophytic compared with low-grade articular chondrocytes agrees with the direction of difference in high-grade compared with low-grade articular chondrocytes for 74% of genes on mRNA level and for 66% of genes on protein level.

In agreement with this, the vast majority of genes with significant mRNA or protein level differences between osteophytic and low-grade articular cartilage also show the same direction of difference in high-grade compared with low-grade articular cartilage (gene expression: 90.1%, protein abundance: 86.5%; both *P* < 10^−15^; supplementary Tables S1 and S2, available at *Rheumatology* online). However, the magnitude of the log-fold-differences between the high- and low-grade articular chondrocytes is smaller (gene expression: 99.5% of 3601 significant genes and 75.4% of all genes, protein abundance: 99.9% of 942 significant proteins and 73.9% of all proteins).

### Gene-level integration across multiple omics levels

#### RNA sequencing and proteomics

There was a significant positive correlation of the differences in gene expression and protein abundance identified between osteophytic and low-grade articular cartilage in the proteomics and the RNA sequencing data (Fig. 1e; Spearman ρ = 0.31, *P* < 10^−15^; based on 4345 genes present in both). We identified 309 genes with significant differences on both RNA and protein level at 0.1% FDR, 88.7% of these differences were directionally concordant (binomial *P* < 10^−15^).

#### Methylation, RNA sequencing and proteomics

We found 56 genes that showed differences in protein abundance and gene expression levels, and also overlapped a DMR between osteophytic and low-grade articular cartilage (Table 1). The direction of change on RNA and protein level agreed for 52 of the 56 genes (binomial *P* < 10^−10^). For all 56 genes, the direction of difference between osteophytic and high-grade articular chondrocytes is the same as the direction of difference between osteophytic and low-grade articular chondrocytes, and 42 genes also have evidence for difference between osteophytic and high-grade articular chondrocytes across all three molecular levels at 5% FDR or lower (Table 1).

**Table 1:** Genes with cross-omics significant differences between osteophytic and low-grade articular chondrocytes

Gene	Gene DMPs in DMRs	Prop BetaFC >0 DMPs	RNA logFC	RNA FDR	Protein logFC	Protein FDR	ENSG	O *vs* H 5% FDR
*ACTB*	4	0	−0.9	9.48E-06	0.34	3.93E-04	075624	
ALDH1A2*	4	1	−2.89	1.08E-07	−3.29	1.44E-09	128918	Y
*ALDH1A3*	10	1	−1.89	3.02E-08	−2.79	2.93E-08	184254	Y
*ARHGDIA*	4	1	−0.48	2.35E-05	−0.37	6.60E-05	141522	Y
*BLVRA*	3	1	−0.63	3.85E-06	−0.33	4.50E-04	106605	Y
*CASP4*	2	0	0.87	1.53E-06	0.61	9.85E-04	196954	
*CHDH*	3	1	−1.68	3.09E-06	−1.32	8.92E-05	016391	Y
*CHI3L2*	4	0.25	−0.84	7.07E-05	−0.97	7.33E-04	064886	Y
*CHST6*	2	1	−0.72	8.75E-05	−0.73	9.80E-05	183196	Y
*CILP*	7	1	−4.44	2.55E-08	−3.55	1.07E-06	138615	Y
COMP*	13	1	−1.16	3.10E-05	−2.72	2.88E-06	105664	Y
*CPPED1*	1	1	−0.9	1.15E-05	−0.69	1.18E-05	103381	Y
*CPT1A*	8	0.625	0.8	9.15E-04	0.91	4.64E-05	110090	
*CSRP1*	5	0	−0.39	5.37E-04	−0.36	3.66E-04	159176	Y
*CYR61*	5	1	−2.09	2.83E-06	−1.82	4.75E-07	142871	Y
*DCN*	3	1	−1.06	1.15E-04	−1.85	8.29E-05	011465	
*EFHD1*	3	1	−2.56	1.79E-06	−0.79	3.98E-04	115468	
*EMILIN1*	2	1	−0.91	3.67E-05	−1.73	5.11E-08	138080	Y
*EMILIN3*	3	1	−2.65	1.15E-06	−2.96	4.11E-06	183798	Y
*FAM162A*	1	0	0.39	1.77E-04	−0.74	1.24E-05	114023	
*FGF1*	14	1	−3.61	9.36E-08	−2.15	1.01E-08	113578	Y
*FIBIN*	3	1	−1.22	1.04E-05	−2.73	1.53E-05	176971	Y
*GALE*	3	1	−1.31	7.84E-06	−1.42	2.54E-08	117308	Y
*GNAS*	2	1	−0.37	4.49E-04	−0.45	5.76E-05	087460	Y
*IDUA*	1	1	0.57	9.82E-05	0.34	9.73E-04	127415	
*IFI16*	5	0	1.67	7.71E-05	1.21	3.55E-06	163565	Y
*IL6*	4	0.5	4.27	2.62E-04	1.61	2.40E-04	136244	Y
*KRT8*	9	1	−2.29	8.55E-09	−2.73	2.54E-08	170421	Y
*MMP13*	4	0	3.16	1.95E-05	2.51	6.43E-08	137745	Y
*NEBL*	3	1	−2.22	1.49E-07	−1.49	8.84E-07	078114	Y
*NME2*	6	1	−0.53	4.07E-04	−0.41	1.05E-04	243678	
*OSBPL10*	4	1	−1.51	1.08E-07	-0.51	2.84E-04	144645	
*OSBPL3*	2	0	1.36	1.19E-05	1.28	6.86E-06	070882	Y
*PAPSS2*	2	1	−0.95	1.82E-06	−0.62	6.16E-05	198682	
*PDLIM4*	3	1	−1.3	4.22E-05	−0.63	1.19E-05	131435	
*PGM1*	1	1	−0.57	2.82E-07	−0.84	4.10E-06	079739	Y
*PRKCZ*	29	1	−1.46	1.56E-06	−0.83	4.52E-05	067606	Y
*PSTPIP1*	3	1	−1.51	2.99E-04	−0.56	2.39E-04	140368	Y
*PTPRE*	2	0.5	−0.77	5.24E-04	−0.74	7.21E-05	132334	Y
*S100A1*	2	1	−1.97	1.75E-05	−1.26	3.61E-05	160678	Y
*SCRN1*	2	1	−0.74	7.21E-05	−0.95	3.52E-05	136193	Y
*SERPINA5*	3	1	−1.76	4.16E-08	−1.58	1.19E-05	188488	Y
*SFN*	6	1	−4.07	1.46E-07	−1.68	3.03E-04	175793	Y
*SH3PXD2B*	5	0	1.28	1.83E-05	0.86	3.39E-04	174705	Y
*SLC25A22*	2	0.5	−0.53	5.48E-04	0.53	1.51E-05	177542	
*SLC29A1*	4	0	−1.28	6.39E-04	−0.58	6.90E-05	112759	Y
*SMOC2*	14	1	−2.09	8.54E-07	−2.29	6.23E-06	112562	Y
*SOD3*	5	1	−1.53	1.20E-06	−1.69	2.42E-05	109610	Y
*TES*	1	1	−1.59	6.30E-07	−1.38	2.16E-07	135269	Y
*TF*	7	1	−4.77	3.16E-09	−0.94	7.82E-04	091513	Y
*TNFAIP2*	8	0.625	1.84	3.75E-05	1.26	1.46E-04	185215	Y
*TPM3*	0	0	−0.5	2.83E-05	1.32	6.05E-04	143549	Y
*TRPV4*	4	1	−0.96	6.16E-04	−0.69	1.65E-04	111199	
*TUBB2B*	5	0	−2.14	8.00E-06	−1.01	3.01E-05	137285	Y
*TYMP*	2	0	1.31	2.96E-04	0.84	4.55E-04	025708	
*UPP1*	5	1	−1.75	8.31E-07	−0.93	1.86E-05	183696	Y

Only genes with significant differences on all three omics levels (methylation, gene expression and protein abundance) are shown. Gene DMPs in DMRs: differentially methylated probes at 0.1% FDR located in DMRs that overlap gene; Prop PosBetaFC DMPs: proportion of gene DMPs in DMRs that show increased methylation in osteophytic cartilage; DMR: differentially methylated region; logFC: log2-fold change (increase means higher value in osteophytic cartilage); FDR: false discovery rate; ENSG: Ensembl gene ID, prefix with ENSG00000; O *vs* H 5% FDR: genes with significant differences between chondrocytes from osteophytic and high-grade articular cartilage across all three molecular levels at 5% or lower FDR (Y = yes).

* Genes associated with OA in genome-wide association studies.

### Link to genetic variants associated with OA

Of the 56 genes that showed significant differences between osteophytic and low-grade articular cartilage on all three molecular levels, two have been robustly associated with OA in published genome-wide association studies. Both also show directionally concordant, significant RNA and protein level differences between osteophytic and high-grade articular chondrocytes at 0.1% FDR, and are contained in DMRs at 5% FDR.

*COMP* demonstrates significantly lower gene and protein levels in osteophytic compared with low-grade articular cartilage, and is located in a hyper-methylated region. The c.1141 G > C (p.Asp369His) missense variant in the gene has been found to significantly increase the risk of OA in a study of hip OA patients who underwent joint replacement surgery [15].

*ALDH1A2* also displays significantly reduced gene and protein levels in osteophytic compared with low-grade articular cartilage, and is located in a hyper-methylated region. Several genetic variants in and close to *ALDH1A2* have been associated with severe hand OA [16]. The most strongly associated variant is rs12907038; the risk allele has been associated with a decrease of *ALDH1A2* gene expression, and another associated variant has also been associated with allelic imbalance in *ALDH1A2* gene expression [16].

We further tested the joint association of all 56 genes with susceptibility to OA using an unpublished dataset from UK Biobank (2396 hip OA cases, 9593 non-OA controls). There was no significant excess of genetic association in the 56 genes together (*P* = 0.2116 using MAGMA, see Methods section).

### Cross-omics pathway analysis

In an integrative comparison of osteophytic and low-grade articular cartilage using all three molecular levels (see Methods section), we identified 36 GO annotations as significantly associated with the molecular changes at 5% FDR (supplementary Table S5, available at *Rheumatology* online). The most significant annotations (Table 2; all FDR < 2%) include gene sets with links to OA (reviewed e.g. in [3]), such as extracellular matrix organization and collagen catabolic process; skeletal system development; inflammatory response; positive regulation of the ERK1 and ERK2 cascade; and platelet aggregation. A further annotation with highly significant association (integrative FDR < 2%) and *P* < 0.05 on each one of the omics levels was endodermal cell differentiation (Table 2).

**Table 2:** Most significant associations identified in the integrative gene set analysis

Gene Ontology annotation	DMR			RNA			Prot			FDR
	*N*	FC	*P*-value	*N*	FC	*P*-value	*N*	FC	*P*-value	
Extracellular matrix organization*	30	1.71	0.0026	40	1.48	0.0049	22	1.90	0.0072	0.0034
Gluconeogenesis	4	0.98	0.71	7	1.13	0.52	19	3.90	0.00001	0.0034
Positive regulation of cytosolic calcium ion concentration	11	1.44	0.081	20	2.45	0.00001	3	1.85	0.24	0.0034
Skeletal system development*	22	2.02	0.0012	28	1.53	0.0028	12	2.27	0.002	0.0034
Inflammatory response	19	1.07	0.40	44	1.89	0.00001	8	1.09	0.43	0.0081
Endodermal cell differentiation*	9	2.55	0.0054	12	2.14	0.0056	6	2.11	0.036	0.011
Positive regulation of ERK1 and ERK2 cascade*	19	1.60	0.014	21	1.53	0.013	11	2.17	0.0042	0.012
Positive regulation of peptidyl-tyrosine phosphorylation	10	1.61	0.079	18	1.94	0.0017	7	2.46	0.0032	0.012
Collagen catabolic process	16	2.27	0.0024	12	1.15	0.32	13	2.46	0.0013	0.012
Platelet aggregation*	8	1.95	0.048	12	1.71	0.027	11	2.71	0.002	0.016

All shown Gene Ontology terms are enriched in the cross-omics analysis at below 2% FDR. DMR: differentially methylated region; RNA: gene expression; Prot: protein abundance*; N*: number of significant genes annotated to GO term; FC: fold-change enrichment; *P*: within-omics empirical *P*-values for enrichment. FDR: integrative false-discovery rate based on combination of the three-omics *P*-values (see Methods section).

* The terms with enrichment *P *< 0.05 across all individual omics analyses.

### Replication of gene expression changes

A previous study [6] examined gene expression differences between osteophytic and low-grade articular cartilage in knee OA patients using a microarray. The authors identified 31 genes with >20-fold change in gene expression and *P* < 0.005. Of these 31 genes, 18 are present in our RNA sequencing data, and all have directionally concordant effects (supplementary Table S6, available at *Rheumatology* online; binomial *P* < 10^−5^). This includes *TF* and *ALDH1A2*, which were identified as significantly different between osteophytic and low-grade articular cartilage on all three omics levels in this study.

## Discussion

This study provides a systematic molecular characterization of osteophytic chondrocytes in OA across genome-wide methylation, gene and protein expression levels. We have shown widespread molecular differences between chondrocytes from osteophytic and low-grade articular OA chondrocytes (as previously seen for gene expression in the knee), with similar but smaller differences between osteophytic and high-grade articular OA chondrocytes. By contrast, there were far fewer significant differences between chondrocytes from high- and low-grade articular cartilage for any given FDR. In a direct comparison, we have shown that the differences between osteophytic chondrocytes and those from low-grade cartilage are positively correlated with the differences between chondrocytes from high- and low-grade articular cartilage. The correlation between these differences is observed despite the morphological and histological dissimilarities between osteophytic and articular tissues. One interpretation would be that, although both tissues are subject to the disease process and the altered internal joint milieu [4], osteophytic chondrocytes are better able to respond in terms of new cartilage production, which may represent attempted joint recovery (e.g. as in the transient phenotype suggested by [6]). Moreover, osteophytes principally develop at the periphery of the articular surface in response to an altered mechanical environment; chondrocyte proliferation is followed by chondrocyte hypertrophy and endochondral ossification within the osteophyte. Several of the genes and pathways identified in this study are implicated in these processes.

Of the 56 genes with differences between osteophytic and low-grade articular cartilage on all three molecular levels, gene expression changes in Transferrin (*TF*), *MMP13* and *ALDH1A2* have also been previously identified in the knee [6], indicating prominent involvement of these genes. *TF* (reduced mRNA and protein levels in osteophytic chondrocytes) is known to be produced in hypertrophic cartilage as a pro-angiogenic molecule [17] and is found in the synovial fluid of OA patients [18]. *MMP13* (increased mRNA and protein levels in osteophytic chondrocytes) is a marker of chondrocyte hypertrophy considered important in cartilage degeneration [19, 20]. *ALDH1A2* is known to have genetic links to OA, discussed in detail below. The 56 genes with significant differences across omics levels also include *CILP* (reduced mRNA and protein levels in osteophytic chondrocytes), which was previously found to be down-regulated in mechanically induced OA in mice [21]; and *IL6* (increased mRNA and protein levels in osteophytic chondrocytes), which has been localized to chondroblasts and preosteoblasts in human osteophytes during endochondral ossification [22]. All of the genes highlighted above also show evidence for differences between chondrocytes from osteophytic and high-grade articular cartilage across all three molecular levels at 5% or lower FDR.

Notably, the 56 genes with differences between chondrocytes from osteophytic and low-grade articular cartilage on all three molecular levels also include two genes that harbor genetic variants robustly associated with OA identified from genome wide association studies, *ALDH1A2* and *COMP*. *ALDH1A2* (aldehyde dehydrogenase 1 family, member A2 or retinaldehyde) is an enzyme that catalyses the synthesis of retinoic acid, the active derivative of vitamin A (retinol). Vitamin A is involved in post-natal bone health and bone remodelling, with both high and low levels having negative effects [23]. *COMP* is a constituent of the cartilage matrix, present in the interterritorial matrix and is involved in collagen fibrillogenesis [24]. Notably, the decreased expression of both genes is consistent across all three molecular levels in this study, and consistent with the molecular mechanisms suggested for the associated genetic variants (reduction of gene expression for *ALDH1A2* and a missense variant in *COMP*). We did not find such cross-omics significant changes of *ALDH1A2* or *COMP* between low- and high-grade articular cartilage (with only protein-level changes *ALDH1A2* significant at 5% FDR), which could suggest that their action is stronger in osteophytic cartilage, or could be due to the limited power in this discovery study. However, we have replicated gene expression changes of *ALDH1A2* in osteophytic cartilage using independent data. Interestingly, the genetic association of *ALDH1A2* was identified in severe hand OA [16], which is characterized by node formation.

Using UK Biobank data, we did not find evidence for association of the joint set of the 56 genes with differences between chondrocytes from osteophytic and low-grade articular cartilage on all three molecular levels. It is possible that some of these genes exhibit molecular changes as a consequence rather than cause of the disease, or are involved in OA progression rather than incidence. The lack of association could also be due to still limited sample size of the genetic data. Larger cohorts will be required to determine the comprehensive set of genetic variants associated with OA, as well as which molecular changes are causal to disease processes.

In the cross-omics GO analysis, several of the gene annotations with highly significant associations have known links to OA: changes in the extracellular matrix, collagen catabolism, inflammation and activity of the ERK cascade are all known interrelated processes taking place in the OA joint [25]. The ERK signalling pathway is important in mesenchymal cell differentiation and can be regulated by mechanical stimuli during joint formation [26, 27]. Another annotation with highly significant cross-omics association (integrative FDR < 2%) was endodermal cell differentiation, which is not directly linked to cartilage, but could reflect tissue development factors involved in osteophyte formation.

We did not find cross-omics differences between osteophytic and articular cartilage in previously reported osteophyte development genes such as *TGFβ*, *PTH* and *IGF1* [4]. This could be explained by the small sample size of the study, or the fact that the chondrocytes were taken from patients with end-stage hip OA, so may not reflect processes involved early in osteophyte development.

The major strengths of this study are the integration of DNA methylation, gene expression and proteomics data, for a comprehensive overview of the changes across molecular levels, and the precise matching of osteophytic, low- and high-grade articular cartilage samples from the same joint. The latter reduces the possibility of false-positives due to biological differences between individuals. This approach has helped illuminate the molecular basis of OA progression; tissue from healthy individuals and early OA stages would be required to characterize the onset of the disease. The main limitation of this study is the size of the cohort examined here. As such, this study is a proof-of-concept discovery study, and replication in larger independent datasets will be required. The deposition of all data in open repositories also allows the data to be combined with other datasets in the future.

As noted above, the molecular associations identified may be a result of, rather than causal to the disease processes. Nonetheless, they can provide insights into characteristics of osteophytic chondrocytes and into disease progression, and suggest targets for further functional follow-up with translational potential.

In summary, we present the first integrative methylation, gene expression and proteomics study across osteophytic and articular cartilage in hip OA. We have identified multiple genes with significant cross-omics changes between osteophytic and low-grade articular cartilage, including two genes associated with OA through genome-wide association studies.

These findings offer evidence that the study of osteophytic cartilage can provide distinct insight into OA pathogenesis.

## Supplementary Material

Supplementary Data
