# Supplementary material for: Widespread epigenomic, transcriptomic and proteomic differences between hip osteophytic and articular chondrocytes in osteoarthritis
Source: Rheumatology (Oxford). 2018 May 8;57(8):1481–9. doi: 10.1093/rheumatology/key101 (PMC6055583; doi:10.1093/rheumatology/key101)
Supplement: Supplementary Data [file key101_suppl_data.zip › rhe-17-1805-File004.docx]

**SUPPLEMENTARY DATA**

**Methods**

**Sample extraction**

*Chondrocyte preparation*

Femoral heads were obtained from patients having total hip replacement surgery following consent under Cambridge University Human Research Tissue Bank National Research Ethics agreement 11/EE/0011.

Specimens were transferred to the laboratory shortly after surgery and the cartilage surface was assessed; that showing a smooth shiny appearance was classified as low-grade, that showing a fibrillated or fissured surface was classified as high-grade and osteophytes were identified as bony protrusions covered with cartilage at the margins of the articular surface (Supplementary Figures 1-2).

The cartilage surface was kept wet with phosphate buffered saline (PBS) and the cartilage within the respective areas was removed with a scalpel down to subchondral bone. Care was taken not to remove tissue below the level of cartilage. This was achieved by cutting into the cartilage at a shallow angle and then parallel to the cartilage surface. Each cartilage portion was weighed, minced with a scalpel and placed in 20ml of Dulbecco’s modified Eagle medium (Invitrogen) containing 10% foetal bovine serum (Invitrogen) and 6 mgml^-1^ collagenase A (Sigma) in a 75cm^2^ tissue culture flask. This was placed on a rotating platform at 55 rpm in a 37°C humidified incubator containing 5% CO_2_ and incubated overnight to digest the cartilage pieces and release the cells. The cell suspension was passed through a 30 μm filter (Miltenyi) and centrifuged at 400 g for 10 min to pellet the cells. These were re-suspended in 1m of PBS and counted on a haemocytometer following 1:1 mixing with trypan blue to determine cell viability

*Histological examination*

A wedge segment from the femoral head containing subchondral bone was removed from a suitable surface showing osteophyte and both high- and low-grade cartilage with underlying subchondral bone. The segments were fixed in 10% paraformaldehyde and subsequently decalcified in formic acid/EDTA solution. They were then embedded in paraffin and sectioned to 5 μm on a rotary microtome. Sections were stained with haematoxylin and eosin viewed on a Leica DM RXA2 microscope fitted with a Surveyor motorised stage (Objective Imaging) and photomicrographs taken with a Q Imaging Retiga EXi camera using Surveyor software.

*Extraction of DNA, RNA, and protein*

DNA, RNA and protein were prepared from isolated cells (1.5 - 8.6 x 10^6^) using the AllPrep kit (Qiagen) according to the manufacturer’s instructions with minor modifications. Briefly, cells were lysed in 350μl RLT buffer containing 3.5μl β-mercaptoethanol and centrifuged in a Qiashredder column (Qiagen) for 2 min at 20,800g. RNA, DNA and protein were purified using a series of column based centrifugation steps and RNA was eluted in 34 μl RNase-free water, DNA in 100 μl EB buffer and the protein pellet solubilised in 100μl 10% sodium dodecyl sulphate in PBS. DNA and RNA preparations were re-precipitated to remove buffer salts and to concentrate the final product: 20μl 3M sodium acetate, pH 5.5 (Ambion) was added per 100μl of DNA or RNA solution and mixed gently. Then, 283μl of molecular grade ethanol (Sigma) was added per 100μl of DNA or RNA solution to precipitate the nucleic acids and the mixture placed at -20°C overnight. Samples were centrigfuged at 20,800g for 30 minutes and the supernatant removed, the pellet was washed with 900μl 70% ethanol and the nucleic acids re-pelleted by spinning at 20,800g for 10 minutes. Nucleic acids were resuspended in 32μl DNase/RNase free water. All samples were stored at -70°C prior to genomic analysis.

**Proteomics**

*Sample processing*Proteins from the hip joint tissue of 9 individuals were quantified by liquid chromatography–mass spectrometry (LC-MS) analyses using isobaric labeling (TMT10plex Thermo Scientific). Protein disulfide bonds were reduced by TCEP and cysteines were blocked with iodoacetamide prior to overnight trypsin digestion. The peptides were labelled with the TMT10plex reagents and the combined mixture was fractionated with high-pH Reversed Phase chromatography (Waters, XBridge C18 column 2.1 x 150 mm, 3.5 μm, 120 Å). For each 10-plex run a commercial control from Promocell (C-14070) plus 3 tissues (low-grade articular, high-grade articular, and osteophytic cartilage) from 3 individuals were included.

*LC-MS Analysis*LC-MS analysis was performed on the Dionex Ultimate 3000 UHPLC system coupled with the Orbitrap Fusion Tribrid Mass Spectrometer (Thermo Scientific). Each fraction was reconstituted in 40 μL 0.1% formic acid and a volume of 7 μL was loaded to the Acclaim PepMap 100, 100 μm × 2 cm C18, 5 μm, 100 Ȧ trapping column at 10 μL/min. The sample was then subjected to a multi-step gradient elution on the Acclaim PepMap RSLC (75 μm × 50 cm, 2 μm, 100 Å) C18 capillary column (Dionex) at 45 °C. Mobile phase (A) was composed of 0.1% formic acid and mobile phase (B) was composed of 80% acetonitrile, 0.1% formic acid. The gradient separation method at flow rate 300 nL/min was as follows: for 90 min gradient to 38% B, for 5 min up to 95% B, for 13 min isocratic at 95% B, re-equilibration to 5% B in 2 min, for 10 min isocratic at 10% B.
Precursors were selected with mass resolution of 120k, AGC 3×10^5^ and IT 100 ms in the top speed mode within 3 sec and were isolated for CID fragmentation with quadrupole isolation width 1.2 Th. Collision energy was set at 35% with AGC 1×10^4^ and IT 35 ms. MS3 quantification spectra were acquired with further HCD fragmentation of the top 10 most abundant CID fragments isolated with Synchronous Precursor Selection (SPS) excluding neutral losses of maximum m/z 18. Iontrap isolation width was set at 0.7 Th for MS1 isolation, collision energy was applied at 55% and the AGC setting was at 5×10^4^ with 80 ms IT. The HCD MS3 spectra were acquired within 110-400 m/z with 60k resolution. Targeted precursors were dynamically excluded for further isolation and activation for 45 seconds with 7 ppm mass tolerance.  The mass spectrometry proteomics data have been deposited to the ProteomeXchange Consortium via the PRIDE partner repository.

*Protein identification and quantification*
The acquired mass spectra were submitted to SequestHT search in Proteome Discoverer 2.1 for protein identification and quantification. The precursor mass tolerance was set at 20 ppm and the fragment ion mass tolerance was set at 0.5 Da for the CID spectra. Spectra were searched for fully tryptic peptides with maximum 2 miss-cleavages and minimum length of 6 amino acids. TMT6plex at N-termimus, K and Carbamidomethyl at C were defined as static modifications. Dynamic modifications included oxidation of M and Deamidation of N,Q. Peptide confidence was estimated with the Percolator node. Peptide FDR was set at 0.01 and validation was based on q-value and decoy database search. All spectra were searched against a UniProt fasta file containing 20,165 reviewed human entries. The Reporter Ion Quantifier node included a custom TMT-10plex Quantification Method with integration window tolerance 15 ppm, integration method the Most Confident Centroid at the MS3 level. Only peptides uniquely belonging to protein groups were used for quantification. TMT intensities were normalised to account for sample loading (the sum of all protein abundances was used as normalisation factor for each sample).

*Analysis for differential abundance*All further computational analyses were carried out in R. Normalised abundance values were transformed to the log2 scale and quantile normalised. We restricted the analysis to 4653 proteins that were quantified in all individuals and tissues across all 3 batches. The limma package (1) in R was used for differential analysis of osteophytic compared to low-grade, and high-grade compared to low-grade articular cartilage. We applied a within-individual paired-sample analysis using the moderated t-statistic from lmfit and eBayes, with a Benjamini-Hochberg FDR to correct for multiple testing.

*Prinicipal component analysis (PCA)*

A principal component analysis was carried out using the prcomp function in R on the normalised abundance values. The analysis was restricted to the proteins with significant differences between osteophytic and low-grade cartilage at 0.1% FDR.

**RNA sequencing**

*Sample processing*

Using Illumina's TruSeq RNA Sample Prep v2 kits, poly-A tailed RNA (mRNA) was purified from total RNA using an oligo dT magnetic bead pull-down. The mRNA was then fragmented using metal ion-catalyzed hydrolysis. A random-primed cDNA library was then synthesised and this resulting double-strand cDNA was used as the input to a standard Illumina library prep: ends were repaired with a combination of fill-in reactions and exonuclease activitiy to produce blunt ends. A-tailing was performed, whereby an "A" base was added to the blunt ends followed by ligation to Illumina Paired-end Sequencing adapters containing unique index sequences, allowing samples to be pooled. The libraries then went through 10 cycles of PCR amplification using KAPA Hifi Polymerase rather than the kit-supplied Illumina PCR Polymerase due to better performance.

Samples were quantified and pooled based on a post-PCR Agilent Bioanalyzer, then the pool was size-selected using the LabChip XT Caliper. The multiplexed library was then sequenced on the Illumina HiSeq 2000, 75bp paired-end read length. Sequenced data was then analysed and quality controlled, and individual cram files were produced.

*Read quantification*
The cram files were converted to bam files using samtools 1.3.1 (2) and then to fastq files using biobambam 0.0.191 (3), after exclusion of reads that failed QC. We obtained transcript-level quantification using salmon 0.7.2 (4) and the GRCh38 transcriptome reference (release 87). All further analysis steps were carried out in R. Gene-level estimated read counts were then obtained using tximport (5), with estimates for 39037 genes based on Ensembl gene IDs. We filtered out genes with estimated count per million <1 in >9 samples, retaining 14029 genes.

*Differential expression*
We used the limma package (1) in R for differential expression analysis of osteophytic vesus low-grade articular, and high-grade versus low-grade articular cartilage. Limma-voom (6) was used to remove heteroscedascity from the estimated count data. We then fitted linear models in the two analyses, with individual ID as a covariate (i.e. using a within-individual paired sample design), applying lmFit and eBayes. We applied a Benjamini-Hochberg FDR to correct for multiple testing.

*Prinicipal component analysis (PCA)*

A principal component analysis was carried out using the prcomp function in R on the estimated filtered log-counts-per-millions. The analysis was restricted to the genes with significant differences between osteophytic and low-grade cartilage at 0.1% FDR.

**Methylation**

*Illumina 450k BeadChip assay*

*Sample submission*

Samples were tested for quality and then quantified to 50ng/ul by the onsite sample management team prior to submission to the Illumina Genotyping pipeline. Before processing begins, manifests for submitted samples are uploaded to Illumina LIMS where each sample plate is assigned an identification batch so that it can be tracked throughout the whole process that follows.

*Bisulfite Conversion*

Before Pre-Amplification sample DNA requires bisulfite conversion using the Zymo EZ-96 DNA Methylation assay. This is completed manually as per Zymo SOP guidelines.

*Pre-Amplification*

Due to the differences in sample plates between the completed Zymo assay and the Illumina assay, pre-Amplification is performed manually following the Illumina MSA4 SOP. Once complete, sample and reagent barcodes are scanned through the Illumina LIMS tracking software. Four micro-litres (200ng) of sample is required (Illumina guidelines) for the pre-Amplification reaction – there is no quantification step after the completion of the Zymo assay.

*Post-Amplification*

Over three days, Post-Amplification (Fragmentation, Precipitation, Resupension, Hybrisation to beadchip and xStaining) processes are completed as per Illumina protocol using four Tecan Freedom Evos. Following the staining process, BeadChips are coated for protection and dried completely under vacuum before scanning commences on five Illumina iScans, four of which are paired with two Illumina Autloader 2.Xs.

*Image Beadchip*

The iScan Control software determines intensity values for each bead type on the BeadChip and creates data files for each channel (.idat). Genomestudio uses this data file in conjunction with the beadpool manifest (.bpm) to analysis the data from the assay.

*Quality Control*

Prior to downstream analysis, all samples undergo an initial QC to establish how successful the assay has performed. Intensity graphs in Genomestudio’s Control Dashboard identify sample performance by measuring dependent and non-dependent controls that are manufactured onto each BeadChip during production.

*Differential methylation*

Idat files from the Illumina 450k BeadChip were parsed and QCed using ChAMP (7); the probe beta values were normalized using the between-sample functional normalization (funnorm) method (8) from the minfi package (9). Differential methylation on the M-values (10) of 426,912 probes post-QC was tested using the CpGassoc package (11), applying a linear model for tissue status at each probe, with individual ID as covariate. Differentially methylated regions (DMRs) were identified using the DMRcate package (12). DMRs were defined as regions with at least 5 differentially methylated probes on the same chromosome with no more than 1kb between adjacent probes.

*Prinicipal component analysis (PCA)*

A principal component analysis was carried out using the prcomp function in R on the M values of methylation probes located in DMRs between osteophytic and low-grade cartilage at 0.1% FDR.

**UK Biobank association analysis**

We used genetic data from the first release of UK Biobank. Genotypes were provided by UK Biobank after initial sample and SNP quality control (QC), phasing, Imputation and principal component analysis were carried out centrally. After imputation, the number of variants reached 73,355,667. We carried out additional QC checks for samples and SNPs. We excluded samples with call rate ≤97%. We checked samples for gender discrepancies, excess heterozygosity, relatedness, ethnicity and we removed possible contaminated and withdrawn samples. We included in 2,396 hospital-diagnosed hip OA cases and controls based on no OA ICD 10 and/or 9 codes, excluding anyone diagnosed with any musculoskeletal disorders, symptoms or signs, such as pain and arthritis. We selected 9,593 controls (approximately 4 times the number of cases). This yielded a final dataset of 16,122,076 variants in 2,396 hospital-diagnosed hip OA cases and 9,593 non-OA controls.

We then carried out a variant-level genetic association analysis, using the likelihood ratio test in SNPTEST v2.5.2 (13) with adjustment for the first 10 Principal Components to control for population structure.

We used MAGMA v1.06 (14) to combine variant association information on gene and on gene-set level. Genotype data of self-reported OA cases and 10,000 controls was used as reference data to estimate LD between SNPs. To test the association of the 56 genes with significant differences between osteophytic and low-grade articular cartilage on all three molecular levels, we used a competitive gene-set analysis. This analysis in MAGMA tested whether genetic variants in the 56 genes are more associated with OA than expected by chance, correcting for the potentially confounding effects of sample size, gene size, gene density and the inverse of the mean minor allele count in the gene, as well as the log of these variables, as recommended.

**Gene Ontology gene-set analysis**

*Individual datasets*

We aimed to test whether particular biological gene sets were enriched among the significant genes from each of the RNA-seq, methylation, and proteomics datasets. We downloaded Gene Ontology (GO) (15) biological process and molecular function gene annotations from Ensembl Biomart on 17 February 2017. We only considered annotations with evidence codes IMP, IPI, IDA, IEP, and TAS. Genes annotated to the same term were treated as a pathway. Only 684 pathways with 20 to 200 genes were considered.

Enrichment was assessed using a 1-sided hypergeometric test and only considering genes with annotations from a particular resource. For example, among the genes 14029 with RNA sequencing data, 7065 genes had GO annotations, and 96 genes were annotated to extracellular matrix organization. Of the 3601 significantly differentially expressed genes at 0.1% FDR, 1985 had GO annotations, and 40 genes were annotated to extracellular matrix organization. Consequently, the enrichment of extracellular matrix organisation genes among the differentially expressed genes was assessed by comparing 40 of 1985 to 96 of 7065 genes. Multiple-testing was accounted for by using a 5% FDR (separately for RNA-seq, methylation, and proteomics data).

Empirical p-values for the enrichments were obtained from randomisations accounting for overlap of significant genes among the RNA-seq, methylation, and proteomics datasets were carried out by subdividing genes into bins as described for integrative analyses below.

*Integrative gene set analyses*

We aimed to integrate the gene sets analyses for the RNA-seq, methylation, and proteomics datasets. We followed the procedure from (16). For each gene set, we asked whether the association across the three datasets (calculated as geometric mean of the *p*-values) was higher than expected by chance. To this end, we obtained 1-sided empirical *p*-values from 100,000 sets of random RNA-seq genes, random methylation genes, and random protein abundance genes. The random sets were chosen to conservatively match the overlap observed among the significant genes as follows. We only considered genes with at least one annotation in the resource.

To jointly construct one set each of random RNA-seq genes, random methylation genes, and random protein abundance genes, we picked: random genes for the overlap of RNA-seq, methylation, and protein abundance; additional random genes for the overlap of RNA-seq and methylation; additional random genes for the overlap of RNA-seq and protein abundance; additional random genes for the overlap of methylation and protein abundance; additional RNA-seq random genes; additional methylation random genes; additional protein abundance random genes.

Random genes were picked to account for gene length as follows. In step 1, we subdivided all genes present in the RNA-seq, methylation, and protein abundance data into 100 bins by increasing exonic length. If the original significant genes in the overlap had *g* genes in a particular bin *b*, we picked *g* random genes from that same bin; this was done for all 100 bins. Steps 2 to 7 were done analogously.

After obtaining the 1-sided empirical *p*-values from the integrative analysis, we used a 5% False-Discovery Rate threshold to correct for multiple testing. We also excluded annotations that were enriched in only one of the three molecular levels (methylation, gene expression, protein abundance), or where fewer than 5 genes contributed to the enrichment on at least two molecular levels (Supplementary Table S5).

**Supplementary Figure S1.** **Three views of a femoral head showing osteophytic cartilage, low-grade and high-grade articular cartilage**


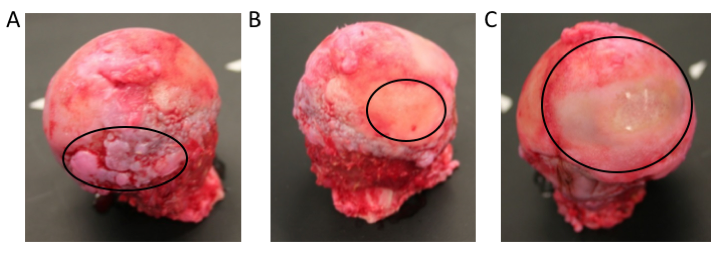


(A) Osteophytic cartilage. (B) Low-grade articular cartilage. (C) High-grade articular cartilage. Each tissue type is highlighted by a black ring.

**Supplementary Figure S2**. **Section of a cartilage bone segment from an osteoarthritic femoral head**


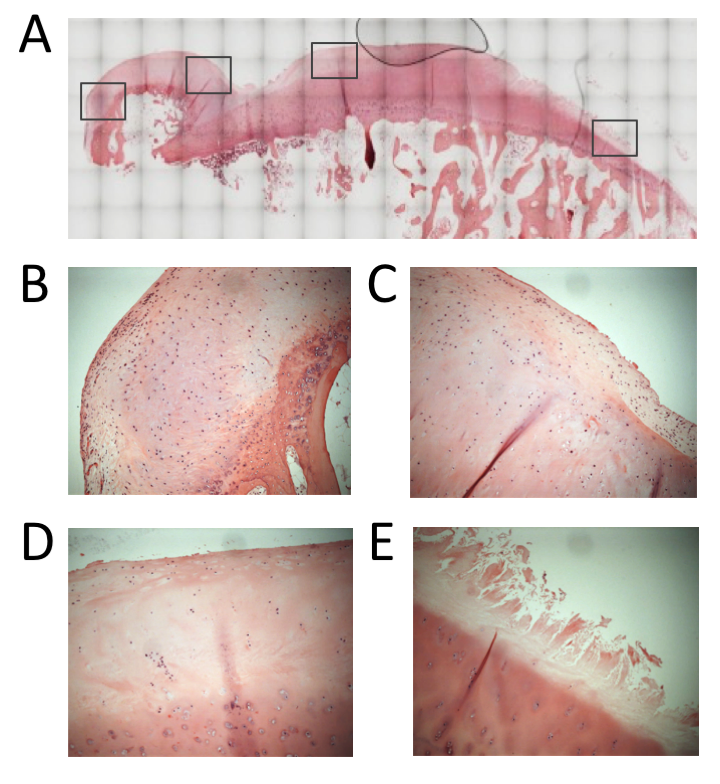


(A) shows an overview of the section, with black rectangles denoting the locations of the regions highlighted in (B-E) from left to right. Osteophytic cartilage is shown in (B, C), low-grade articular cartilage in (D), and high-grade articular cartilage in (E).

**Supplementary Table S1. Protein abundance differences between osteophytic and low-grade cartilage, and between high-grade and low-grade cartilage**

Columns with name suffix _OvsL denote comparison of osteophytic to low-grade cartilage; columns with name suffix _HvsL denote comparison of high-grade to low-grade cartilage; columns with name suffix _OvsH denote comparison of osteophytic to high-grade cartilage.
logFC: log-fold-change; CI.L: lower bound of 95% confidence interval for log-fold-change; CI.U: upper bound of 95% confidence interval for log-fold-change; AveExpr: average expression value; t: moderated t-test statistic from limma; P.Value: p-value for moderated t-test statistic; ENSG: Ensembl gene ID (only where unique mapping possible).

**Supplementary Table S2. Gene expression differences between osteophytic and low-grade cartilage, and between high-grade and low-grade cartilage**

ENSG: Ensembl gene. Columns with name suffix _OvsL denote comparison of osteophytic to low-grade cartilage; columns with name suffix _HvsL denote comparison of high-grade to low-grade cartilage; columns with name suffix _OvsH denote comparison of osteophytic to high-grade cartilage.
logFC: log-fold-change; CI.L: lower bound of 95% confidence interval for log-fold-change; CI.U: upper bound of 95% confidence interval for log-fold-change; AveExpr: average expression value; t: moderated t-test statistic from limma; P.Value: p-value for moderated t-test statistic; adj.P.Val: FDR; GeneName: gene name (only where unique mapping possible).

**Supplementary Table S3. Differentially methylated regions between osteophytic and low-grade cartilage at 0.1% FDR**

chr: chromosome; start: start of region; end: end of region; DMR: unique DMR identified; minfdr: estimated minimum FDR at which the region is significant; maxbetafc: maximal absolute fold-change of methylation for any probe within the DMR; meanbetafc: mean absolute fold-change of methylation for any probe within the DMR; no.cpgs: number of probes within region; n_sig_probe_level: number of probes within DMR that are significant at 0.1% FDR in the probe-level analysis; prop_pos: proportion of probes within DMR that have increased methylation in osteophytic cartilage.

**Supplementary Table S4.** **Differentially methylated regions between osteophytic and high-grade cartilage at 0.1% FDR**

chr: chromosome; start: start of region; end: end of region; DMR: unique DMR identified; minfdr: estimated minimum FDR at which the region is significant; maxbetafc: maximal absolute fold-change of methylation for any probe within the DMR; meanbetafc: mean absolute fold-change of methylation for any probe within the DMR; no.cpgs: number of probes within region; n_sig_probe_level: number of probes within DMR that are significant at 0.1% FDR in the probe-level analysis; prop_pos: proportion of probes within DMR that have increased methylation in osteophytic cartilage.

**Supplementary Table S5. Gene Ontology enrichment results for the integrative cross-omics analysis of differences between osteophytic and low-grade articular cartilage**

Annotation; Gene Ontology annotation; FishCombStat: combined statistic for the cross-omics enrichment (see Methods); EmpPOneSided: one-sided empirical p-value for the cross-omics enrichment; EmpPFDR: FDR for the EmpPOneSided; PassEnrichQC: 1 if the enrichment passes quality control (see Methods), 0 otherwise.

For the individual –omics results, columns with name suffix _Prot refer to proteomics, with _RNA to RNAseq, with _Methyl to DMR data. ListPathGenes: genes with annotation among significant genes; ListGenes: total of significant genes with any annotation; PathGenes: genes with this annotation that were assayed in the –omics experiment; TotGenes: total of genes with any annotation that were assayed in the –omics experiment; Enrichm: fold-change enrichment of annotation among significant genes; HyperGeomEnrichmP: 1-sided hypergeometric p-value for enrichment; EmpP: empirical 1-sided p-value for enrichment.

**Supplementary Table S6. Replication results using published microarray gene expression data for osteophytic and low-grade articular cartilage**

Gelse 2012: results from (17); RNA: results from gene expression analysis in this study; Proteomics: results from protein abundance analysis in this study. GeneBank accession: GeneBank accession number of probe used to measure gene in (17); Fold-change: fold-change in osteophytic compared to low-grade cartilage; P-value: p-value for change; FDR: false-discovery rate. Of the 18 genes present in our RNAseq data, all have directionally concordant effects (some other genes could be missing due to changes in gene name since 2012 or non-unique gene names, which we excluded).

**References**

1. Ritchie ME, Phipson B, Wu D, Hu Y, Law CW, Shi W, et al. limma powers differential expression analyses for RNA-sequencing and microarray studies. Nucleic acids research. 2015;43(7):e47.

2. Li H, Handsaker B, Wysoker A, Fennell T, Ruan J, Homer N, et al. The Sequence Alignment/Map format and SAMtools. Bioinformatics. 2009;25(16):2078-9.

3. Tischler G, Leonard S. biobambam: tools for read pair collation based algorithms on BAM files. Source Code for Biology and Medicine. 2014;9:13.

4. Patro R, Duggal G, Love MI, Irizarry RA, Kingsford C. Salmon provides accurate, fast, and bias-aware transcript expression estimates using dual-phase inference. bioRxiv. 2016.

5. Soneson C, Love M, Robinson M. Differential analyses for RNA-seq: transcript-level estimates improve gene-level inferences [version 1; referees: 2 approved]; 2015.

6. Law CW, Chen Y, Shi W, Smyth GK. voom: precision weights unlock linear model analysis tools for RNA-seq read counts. Genome Biology. 2014;15(2):R29.

7. Morris TJ, Butcher LM, Feber A, Teschendorff AE, Chakravarthy AR, Wojdacz TK, et al. ChAMP: 450k Chip Analysis Methylation Pipeline. Bioinformatics. 2014;30(3):428-30.

8. Fortin J-P, Labbe A, Lemire M, Zanke BW, Hudson TJ, Fertig EJ, et al. Functional normalization of 450k methylation array data improves replication in large cancer studies. Genome Biology. 2014;15(11):503.

9. Aryee MJ, Jaffe AE, Corrada-Bravo H, Ladd-Acosta C, Feinberg AP, Hansen KD. Minfi: A flexible and comprehensive Bioconductor package for the analysis of Infinium DNA Methylation microarrays. Bioinformatics. 2014;30.

10. Du P, Zhang X, Huang C-C, Jafari N, Kibbe WA, Hou L, et al. Comparison of Beta-value and M-value methods for quantifying methylation levels by microarray analysis. BMC Bioinformatics. 2010;11(1):587.

11. Barfield RT, Kilaru V, Smith AK, Conneely KN. CpGassoc: an R function for analysis of DNA methylation microarray data. Bioinformatics. 2012;28(9):1280-1.

12. Peters TJ, Buckley MJ, Statham AL, Pidsley R, Samaras K, V Lord R, et al. De novo identification of differentially methylated regions in the human genome. Epigenetics & Chromatin. 2015;8(1):6.

13. Marchini J, Howie B. Genotype imputation for genome-wide association studies. Nat Rev Genet. 2010;11(7):499-511.

14. de Leeuw CA, Mooij JM, Heskes T, Posthuma D. MAGMA: Generalized Gene-Set Analysis of GWAS Data. PLoS Comput Biol. 2015;11(4):e1004219.

15. Ashburner M, Ball CA, Blake JA, Botstein D, Butler H, Cherry JM, et al. Gene ontology: tool for the unification of biology. The Gene Ontology Consortium. Nat Genet. 2000;25(1):25-9.

16. Steinberg J, Ritchie GRS, Roumeliotis TI, Jayasuriya RL, Clark MJ, Brooks RA, et al. Integrative epigenomics, transcriptomics and proteomics of patient chondrocytes reveal genes and pathways involved in osteoarthritis. Sci Rep. 2017;7(1):8935.

17. Gelse K, Ekici AB, Cipa F, Swoboda B, Carl HD, Olk A, et al. Molecular differentiation between osteophytic and articular cartilage – clues for a transient and permanent chondrocyte phenotype. Osteoarthritis Cartilage. 2012;20(2):162-71.
